# Intrinsic Expression of Immune Checkpoint Molecule TIGIT Could Help Tumor Growth *in vivo* by Suppressing the Function of NK and CD8^+^ T Cells

**DOI:** 10.3389/fimmu.2018.02821

**Published:** 2018-11-29

**Authors:** Xiu-Man Zhou, Wan-Qiong Li, Ya-Hong Wu, Lu Han, Xin-Guang Cao, Xuan-Ming Yang, Hong-Fei Wang, Wen-Shan Zhao, Wen-Jie Zhai, Yuan-Ming Qi, Yan-Feng Gao

**Affiliations:** ^1^School of Life Sciences, Zhengzhou University, Zhengzhou, China; ^2^Cancer Biotherapy Center, Henan Cancer Hospital, Zhengzhou, China; ^3^Department of General Surgery, Henan Cancer Hospital, Zhengzhou, China; ^4^School of Life Science and Biotechnology, Shanghai Jiao Tong University, Shanghai, China

**Keywords:** TIGIT, CD8^+^ T cell, colorectal cancer, cancer immunotherapy, immune checkpoint blockade

## Abstract

TIGIT, an immune checkpoint molecule widely expressed on NK cells, activated T cells and Tregs, has been involved in delivering inhibitory signals through the interaction with PVR. The blockade of TIGIT/PVR interaction is a promising approach in cancer immunotherapy. Here, we unexpectedly discovered the expression of TIGIT in murine tumor cells. To elucidate the mechanism of such intrinsic expression, TIGIT knockout murine colorectal CT26 and MC38 cell lines were generated by using CRISPR/Cas9 system. Although TIGIT knockout showed no effects on proliferation and colony formation of tumor cells *in vitro*, the tumor growth in mice was considerably inhibited. TIGIT knockout led to the increase of IFN-γ secretion by NK and CD8^+^ T cells. Further, in BABL/c nude mice, CD8^+^ T cells depleting mice and NK cells depleting nude mice, the promotion of tumor growth was significantly diminished, suggesting that both NK cells and CD8^+^ T cells were involved in the tumor promoting process mediated by intrinsic TIGIT. In addition, blocking TIGIT/PVR interaction by the antibody or recombinant PVR protein could elicit anti-tumor effects by facilitating the tumor infiltration and restoring the function of CD8^+^ T cells, and the antibody-mediate TIGIT blockade could inhibit MC38 tumor growth through blocking TIGIT expressed on tumor cells. We therefore propose a novel TIGIT/PVR interaction mode that tumor intrinsic TIGIT delivers inhibitory signals to CD8^+^ T cells and NK cells by engaging with PVR.

## Introduction

More and more work from both clinical and basic research revealed that immune checkpoint blockade, especially CTLA-4 and PD-1/PD-L1, could successfully reinvigorate T cell function to fight against cancer (1–6). Antibodies targeting PD-1 or PD-L1 have exhibited persistent clinical benefits with the response rates of approximately 30–40% in patients with advanced cancers (7–9). Application of immune checkpoint antibodies, alone or in combination, has also achieved great success in manipulating non-melanoma cancers such as non-small-cell lung cancer (NSCLC), metastatic renal cell carcinoma (RCC), bladder cancer and Hodgkin's lymphoma (10–15). However, PD-1 or PD-L1 blockade led to very limited response rate in colorectal cancer patients, only those with microsatellite instability (MSI) might get clinical benefits (16–18). Therefore, more potential therapeutic targets or strategies are still urgently needed, such as novel immunotherapeutic targets or combinations.

T cell immunoglobulin and immunoreceptor tyrosine-based inhibitory motif (TIGIT) is a novel co-inhibitory receptor widely expressed on activated T cells, NK cells, memory T cells, follicular Th cells and Tregs (19–21). Similar to the well-defined CD28/CTLA-4 pathway, TIGIT competes with its co-stimulatory counterpart CD226 to bind toward the conjunct ligands CD155 (also known as PVR) and CD112 (also known as PVRL2) (22). Ligation of PVR with TIGIT mediates inhibitory signals to T cells, but transmits stimulatory signals while binds to CD226 (23). TIGIT can not only indirectly inhibit T cell response by triggering PVR on DCs, thereby inducing the production of IL-10 and preventing DC maturation, but also directly exert T cell intrinsic inhibitory effects via recruitment of the phosphatases SHIP1 and SHP2 (20, 24). Additionally, TIGIT/PVR ligation also leads to a sharp reduction of NK cytotoxicity, granule polarization, and cytokine release (25). Recent studies revealed that TIGIT/PVR signaling had also been implicated in inhibiting the metabolism of CD8^+^ T cells, and therefore suppressing the effector function (26).

It has been proven that leveraging TIGIT in combination with other modalities such as PD-1/PD-L1 blockade may achieve robust clinical outcomes. Chauvin JM and colleagues confirmed that TIGIT and PD-1 co-blockade could improve the expansion and function of circulating tumor specific CD8^+^ T cells and tumor infiltrating CD8^+^ T cells thus enhancing CD8^+^ T cell responses to melanoma. And for patients with advanced melanoma, this combination could improve the clinical efficacy of PD-1 blockade (27). Anderson et al. demonstrated that TIGIT and TIM-3 were co-expressed on Tregs and acted synergistically to drive suppressive tumor microenvironment. Besides, in TIGIT^−/−^ mice, the synergistic effects of TIM-3 blockade depended on CD8^+^ T cells (28). TIGIT blockade alone or synergized with PD-L1 blockade could re-activate CD8^+^ T cells in the draining lymph nodes to reject transplanted tumors (29). Currently, anti-TIGIT (MTIG7192A) combined with anti-PD-L1 (atezolizumab) is being evaluated in a clinical trial involving locally advanced or metastatic tumors (NCT02794571). Despite these findings, it remains largely unknown about how TIGIT modulates the tumor microenvironment, and also whether it is a promising therapeutic target in colorectal cancer.

Here, we found TIGIT was over-expressed in colon cancer tissues compared with adjacent normal tissues. To study the function of TIGIT, we examined the expression in murine colon cancer cell lines, and unexpectedly discovered its intrinsic expression. The function of intrinsic TIGIT in colon cancer cells was investigated by using the CRISPR/Cas9 knockout model. TIGIT knockout showed no effect on the proliferation and colony formation of colorectal cancer cells *in vitro*, but could remarkably decrease tumor growth in mice. To elucidate the suppressed effects of TIGIT on the immune cells of the tumor-bearing mice, the role of NK cells and CD8^+^ T cells was studied by specific antibody depletion strategy. In addition, the anti-tumor effects of blocking TIGIT by the antibody or recombinant PVR protein were investigated, and the anti-tumor effects of anti-TIGIT through tumor intrinsic TIGIT were also studied. Our results broaden the knowledge that TIGIT could not only express on immune cells, but also on cancer cells, and this intrinsic expression may deliver inhibitory signals through the interaction with PVR on CD8^+^ T cells and NK cells.

## Materials and methods

### Gene expression analysis

RNA-seq data from colon cancer and matched normal tissue samples were obtained from dataset GSE37182 in Gene Expression Omnibus (GEO) database. Processing and analysis of TIGIT and PD-1 expression profile was performed using the R language.

### Tumor specimens

Tumor and matched peri-tumor tissue specimens (*n* = 9) were collected from the same patients with colorectal tumors. The peri-tumor tissues were at least 5 cm away from the visible tumor mass as previously described (30). Tissue specimens were cut into small pieces, cells were dissociated by frosted slides and filtered through a 70-μm nylon cell strainer to remove large chunks of tissue. Single cell suspensions were stained with certain antibodies for flow cytometry analysis. Tissue specimens were obtained from Henan Cancer Hospital, Affiliated of Zhengzhou University (Zhengzhou, China) with the approval of the Institutional Ethics Review Board.

### Antibodies and reagents

Anti-human CD45 FITC (HI30), anti-human TIGIT APC (MBSA43), anti-human PD-1 PE (MIH4), anti-mouse TIGIT PE (GIGD7), anti-mouse PVR APC (TX56), anti-mouse PD-1 PE (J43), anti-mouse PD-L1 PE (MIH5), anti-mouse CD45 FITC (30-F11), anti-mouse CD3 PerCP-eFluor710 (17A2), anti-mouse CD8α PE (53-6.7) anti-mouse CD49b PE/APC (DX5), anti-mouse CD19 APC (eBio1D3), anti-mouse CD11c APC (N418), anti-mouse CD11b APC (M1/70), anti-mouse Ly-6G(Gr-1) PE- Cyanine7 (RB6-8C5), anti-mouse F4/80 PerCP-Cyanine5.5 (BM8), anti-mouse IFN-γ APC (XMG1.2), mouse IgG1κ isotype control (P3.6.2.8.1), rat IgM isotype control (eBR2M), rat IgG2α κ isotype control (eBR2a) and rat IgG1 κ isotype control (eBRG1) antibodies were purchased from eBioscience. Anti-mouse TIGIT APC (1G9) was purchased from BioLegend. Antibodies anti-asialo-GM1 (catalog 986-10001) (Wako Chemicals GmbH, Germany) and rabbit IgG control (I8140) (Sigma) were used for NK cell depletion. EasySep mouse CD8^+^ T cell isolation kit (catalog 19853) and EasySep mouse NK cell isolation kit (catalog 19855) (STEMCELL) were used for cell sorting.

### Cell lines and cell culture

Murine colorectal cancer cell lines CT26 and MC38 were cultured in DMEM medium (GIBCO, Grand Island, USA) supplemented with 10% FBS (BI, USA), 100 U/mL penicillin (Solarbio, China), 100 U/mL streptomycin (Solarbio, China) at 37°C with 5% CO_2_ under fully humidified conditions. Murine breast cancer cell line 4T1, Lewis lung carcinoma cell line, melanoma cell lines B16 and B16-F10 were cultured in RPMI 1640 medium (GIBCO, Grand Island, USA).

### RNA isolation and RT-PCR

Total RNA from cells was extracted by E.Z.N.A.® Total RNA Kit II (Omega, USA) and reversely transcripted into cDNA using the RevertAid cDNA synthesis kit (Thermo Scientific, USA) according to the manufacturer's instruction. Polymerase Chain Reaction was performed with the primers: 5′-ATGGTGAAGGTCGGTGTGA-3′ and 5′-TTACTCCTTGGAGGCCATGTA-3′ for mouse GAPDH; 5′-ATGCATGGCTGGCTGCTCCT-3′ and 5′-CCCTTAGCCAGTCTTCGATACAGC-3′ for mouse TIGIT; 5′-ATGTGGGTCCGGCAGGT-3′ and 5′-TCAAAGAGGCCAAGAACAATGTC-3′ for mouse PD-1.

### CRISPR/Cas9 knockout (KO) cell lines

TIGIT knockout CT26 and MC38 cells were established using a CRISPR/Cas9 system according to the standard protocol provided by Zhang's lab (31). Briefly, single guide RNA (sgRNA) was designed using online CRISPR Design Tool (http://tools.genome-engineering.org) and cloned into plasmid lentiCRISPRv2 (catalog 52961). The sgRNA sequences of mouse TIGIT were 5′-GCTGAAGTGACCCAAGTCGAC-3′ for sgRNA1, 5′-GTTCAGTCTTCAGTGATCGGG-3′ for sgRNA2. Lentivirus was produced with the packing vector pCMV-VSV-G (catalog 8454), and psPAX2 (catalog 12260) in 293T cells. TIGIT knockout CT26 and MC38 cell lines CT26-sgRNA1, CT26-sgRNA2, MC38-sgRNA1 and MC38-sgRNA2 were established by puromycin selection following lentivirus infection.

### MTT assay

The proliferation of TIGIT knockout CT26 and MC38 cells were determined by MTT assay. Briefly, cells were seeded into a 96-well plate at a density of 3,000 cells/well in DMEM medium supplemented with 10% FBS. After 24, 48, and 72 h, cell viability was detected using MTT reagent (Sigma, USA) dissolved in PBS 7.2 (5 mg/mL) and incubated at 37°C for 4 h. After removing incubation medium, formazan crystals were dissolved in 150 μL DMSO. MTT reduction was quantified by measuring the absorbance at 490 nm.

### Soft agar colony formation assay

In a 6-well plate, 0.6% agarose in DMEM medium containing 8,000 cells/well was plated on the top of a solidified layer of 1.25% agarose. After 2 weeks, the colonies were stained with crystal violet (0.01%), washed with PBS, then imaged and analyzed using the Image J software.

### Tumor model and treatments

All mice were bought from Vital River Laboratory (Beijing, China) and maintained in a specific pathogen-free facility. Six-week-old female BALB/c mice or nude mice were subcutaneously injected on the right back with 1 × 10^5^ syngeneic CT26 cells, or the TIGIT KO cells (CT26-sgRNA1 or CT26-sgRNA2). C57BL/6 mice were subcutaneously inoculated with 5 × 10^5^ MC38 cells or the TIGIT KO cells (MC38-sgRNA1 and MC38-sgRNA2), for antibody treated model 1 × 10^6^ MC38 cells were inoculated.

For depletion of indicated cell population, BALB/c mice were injected with 250 μg anti-asialo-GM1 depleting antibody, 200 μg CD8-depleting antibody (clone: YTS 169.4), 250 μg CD4-depleting antibody (clone: GK1.5) or matched isotype control antibodies the day before tumor cell inoculation and every 4 days thereafter (32–34). Depletion efficiency was verified by flow cytometry.

One week post tumor cell inoculation, mice bearing tumors of 50–100 mm^3^ were grouped randomly and then treated with anti-TIGIT (200 μg; 1G9; BioXCell, USA), recombinant mouse PVR protein (200 μg) or isotype control antibody (mouse IgG) by intraperitoneal injection every 3 days for 2 weeks. For recombinant mouse PVR protein, the amount of endotoxin was determined to be < 0.2 EU/mg (limit of detection).

Tumor sizes were measured using a digital caliper, and tumor volumes were calculated as V = 1/2 × a (length) × b (width) × c (height).

### Intracellular cytokine staining assay

Single cell suspension of mouse spleen or draining lymph node was prepared by gentle mechanical disruption. NK cells and CD8^+^ T cells were obtained from spleen or draining lymph node cell suspensions via negative enrichment, according to the manufacturer's protocol (STEMCELL). Enriched NK cells and CD8^+^ T cells were cultured in complete DMEM medium. Tumor-infiltrating lymphocytes were isolated from tumor cell suspension by discontinuous Percoll density gradients (40 and 70%) (GE Healthcare). Cells were stimulated with 20 ng/mL phorbol 12-myristate 13-acetate (PMA, Sigma) and 1 μM ionomycin (Sigma) in the presence of protein transport inhibitor cocktail (eBioscience) for 4 h. Cells were then stained with surface markers antibodies anti-mouse CD8α PE (53-6.7) or anti-mouse CD49b PE (DX5) prior to fixation and permeabilization. Permeabilized cells were then stained with anti-mouse IFN-γ APC antibody (XMG1.2) (eBioscience) or isotype control.

For cell staining, cells were harvested and suspended in 50 μL PBS containing 0.5% fetal bovine serum, incubated with the corresponding fluorochrome conjugated antibodies at 4°C for 30 min, washed, and analyzed by a FACS Calibur (BD Bioscience) flow cytometry.

### Statistical analysis

The data were shown as means ± SEM unless otherwise indicated. Statistical analysis was conducted with one-tailed and paired 2-tailed Student's *t*-test for differences between groups. *p* < 0.05, *p* < 0.01, and *p* < 0.001 were considered statistically significant.

## Results

### TIGIT was overexpressed in colorectal tumor tissue and intrinsically expressed on tumor cells

**Figure 1:**
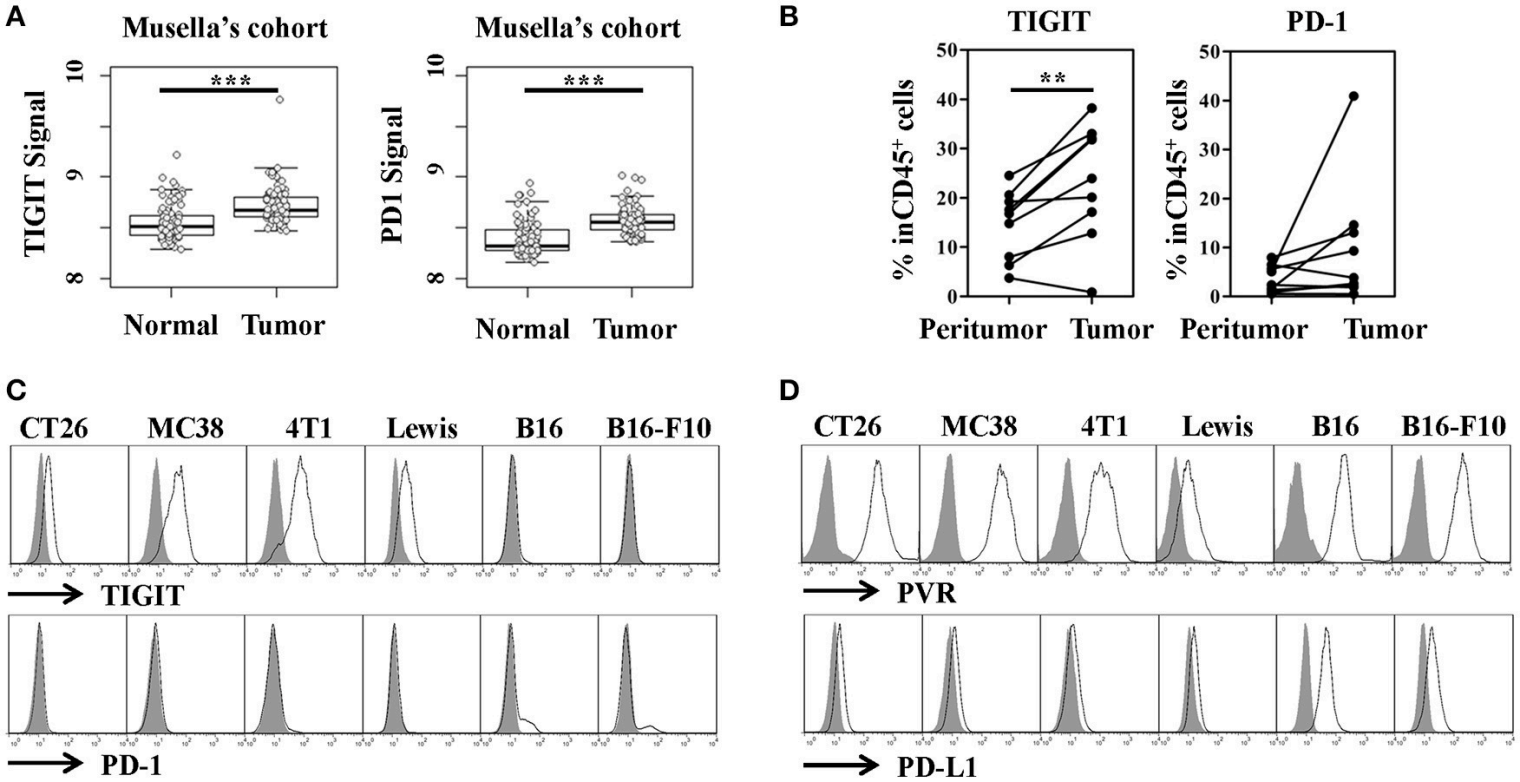
TIGIT and PD-1 expression by tumor tissues and cell lines. **(A)** TIGIT and PD-1 were highly expressed in colon cancer dataset of Musella's cohort (GSE37182). Statistical significance was determined by Student's *t*-test (^***^p < 0.001). **(B)** The percentage of TIGIT^+^ or PD-1^+^ cells of the total CD45 cell population in peri-tumor and tumor from patients with colorectal cancer (*n* = 9). Statistical significance was determined by paired 2-tailed Student's *t*-test. (^**^p < 0.01). **(C)** Flow cytometry analysis of TIGIT and PD-1 expression on murine tumor cell lines, the gray-shaded histogram represents the isotype control. **(D)** Flow cytometry analysis of PVR (TIGIT ligand) and PD-L1 (PD-1 ligand) on murine tumor cell lines, the gray-shaded histogram represents the isotype control.

To study whether TIGIT could serve as a potential target in colorectal cancer, we analyzed the expression of TIGIT in colorectal cancer patients of Musella's cohort (GSE37182). By plotting TIGIT expression in tumor and adjacent normal tissues, respectively, we found that TIGIT was overexpressed in tumors compared to normal tissues (Figure 1A, left panel, tumor vs. normal, *p* < 0.001). Meanwhile, we also observed that PD-1 was significantly overexpressed in tumor tissues (Figure 1A, right panel, tumor vs. normal, *p* < 0.001). We next examined TIGIT and PD-1 expression in fresh tumor samples from patients with colorectal cancer, and noticed that TIGIT was highly expressed on CD45^+^ cells in colorectal cancer samples (Figure 1B, left panel, tumor vs. peri-tumor, *p* < 0.01). Although TIGIT expression occurs mostly on CD45^+^ immune cells, the obvious expression of TIGIT was observed on CD45^−^ cells in some cases. (Figure S1).

It's reported that the function of TIGIT mainly built on its expression on natural killer (NK) cells, activated T cells and regulatory T cells (Tregs), which limited the further understanding of this inhibitory immune checkpoint molecule. To elucidate the function and therapeutic value of TIGIT in colorectal cancer, its expression in murine colorectal cancer cells was detected and compared with that of PD-1. Unexpectedly, we firstly discovered that TIGIT was expressed in mRNA level in murine colorectal cancer cell lines CT26 and MC38. To verify this finding, breast cancer cell line 4T1, Lewis lung carcinoma cell line and melanoma cell lines B16 and B16-F10 were also examined, and TIGIT mRNA was detected in these cells as well. On the other hand, PD-1 mRNA expression was only detected in B16 and B16-F10, but not other cell lines (Figure S2). Further, we determined the expression of TIGIT and PD-1 in protein level by flow cytometry. Consistent with the previous report (35), PD-1 only expressed on a small fraction of the melanoma cell lines B16 and B16-F10, but TIGIT was expressed in all these murine cell lines mentioned above (Figure 1C). We confirmed the results by analyzing TIGIT protein expression with another antibody (Clone: 1G9) (Figure S3). PVR and PD-L1, as the corresponding ligands of TIGIT and PD-1, respectively, were also expressed in these murine cancer cell lines (Figure 1D). Although most of the current studies suggested that TIGIT might function similarly as PD-1, the expression profile of TIGIT was much different from PD-1. The widely expression of TIGIT in tumor cells also suggested the distinct function involved.

### Knockout of TIGIT in colorectal cancer cells did not impact cell proliferation and colony formation

**Figure 2:**
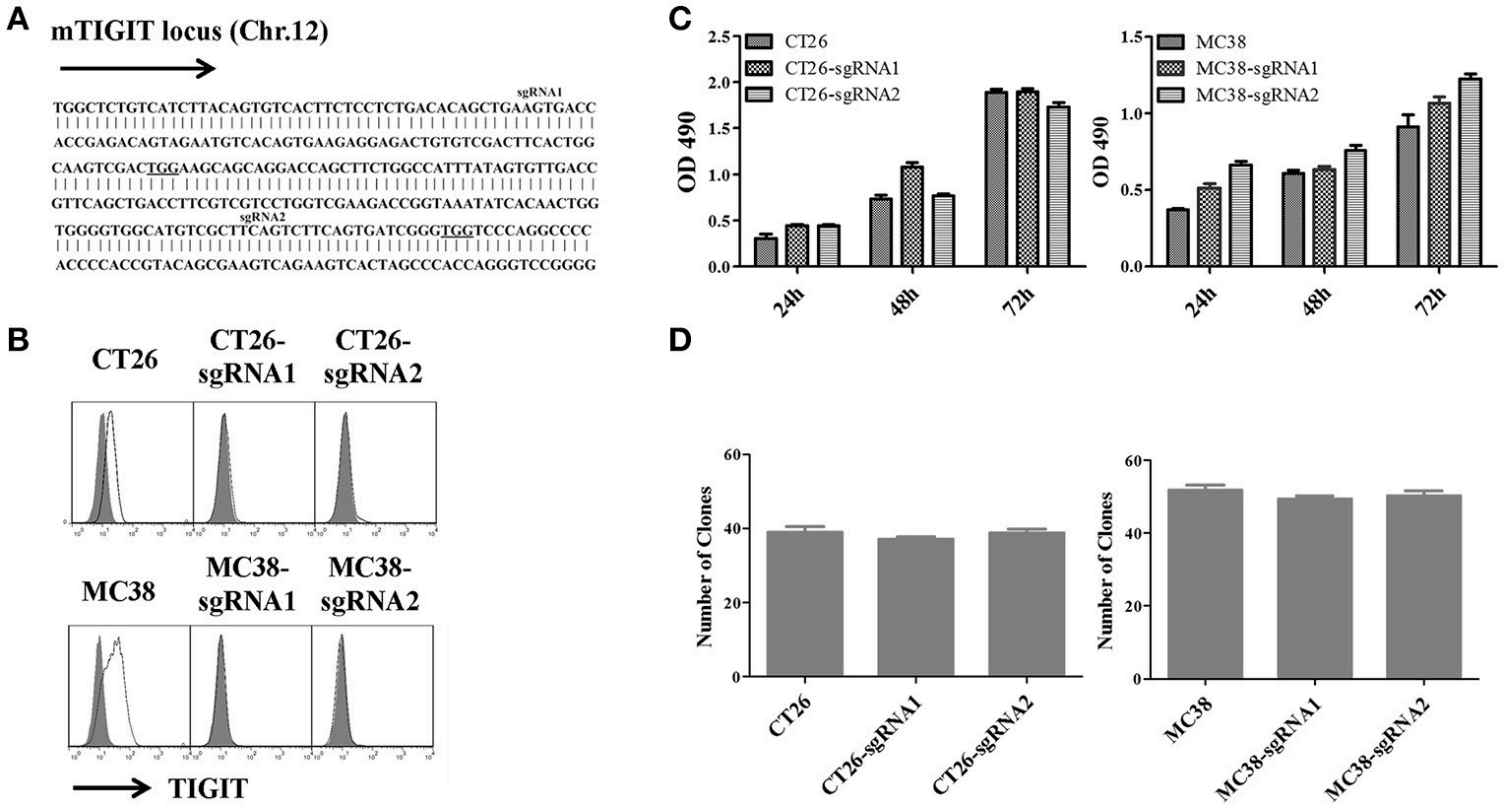
Knockout of TIGIT in colorectal cancer cells could not affect cell proliferation and colony formation. **(A)** Schematic diagram of two sgRNAs targeting mouse TIGIT. The sgRNAs targeting sites on the sense strand are shown in bold. PAM sequences are underlined. **(B)** Flow cytometry analysis of TIGIT expression on the TIGIT knockout (KO) CT26 and MC38 cell lines vs. corresponding parental cell lines, stained with anti-TIGIT (solid lines), the gray-shaded histogram represents the isotype control. Cell proliferation **(C)** and colony formation (**D**) of TIGIT knockout (KO) CT26 and MC38 cell lines vs. corresponding parental cell lines. Cells were seeded into 96-well culture plates at a density 3,000 cells/well. After 24, 48, and 72 h, cell viability was measured using MTT reagent and DMSO. MTT reduction was quantified by measuring the absorbance at 490 nm (OD = optical density). Cells were seeded into 6-well culture plates at a density 8,000 cells/well. Two weeks later, the colonies were stained by 0.2% crystal violet and counted in different fields of view (*n* = 30).

To investigate the potential role of TIGIT in tumor cells, we established stable TIGIT knockout (KO) colorectal cancer cell lines using CRISPR/Cas9 system. We designed two single guide RNAs named sgRNA1 and sgRNA2 to target different exons of TIGIT (Figure 2A). After being transfected with lentivirus packaged in HEK-293T, the corresponding TIGIT knockout CT26 and MC38 cells were established. The expression of TIGIT was verified by FACS analysis (Figure 2B). The expression of PVR and PD-L1 was not affected by TIGIT knockout (Figure S4). To evaluate the effects of TIGIT knockout on these tumor cells, MTT and soft agar assays were conducted. TIGIT knockout showed no significant effects on proliferation (Figure 2C) and colony formation (Figure 2D) when compared with the parental CT26 and MC38 cells.

### TIGIT knockout remarkably impaired the tumorigenicity of murine colorectal cancer cells *In vivo*

**Figure 3:**
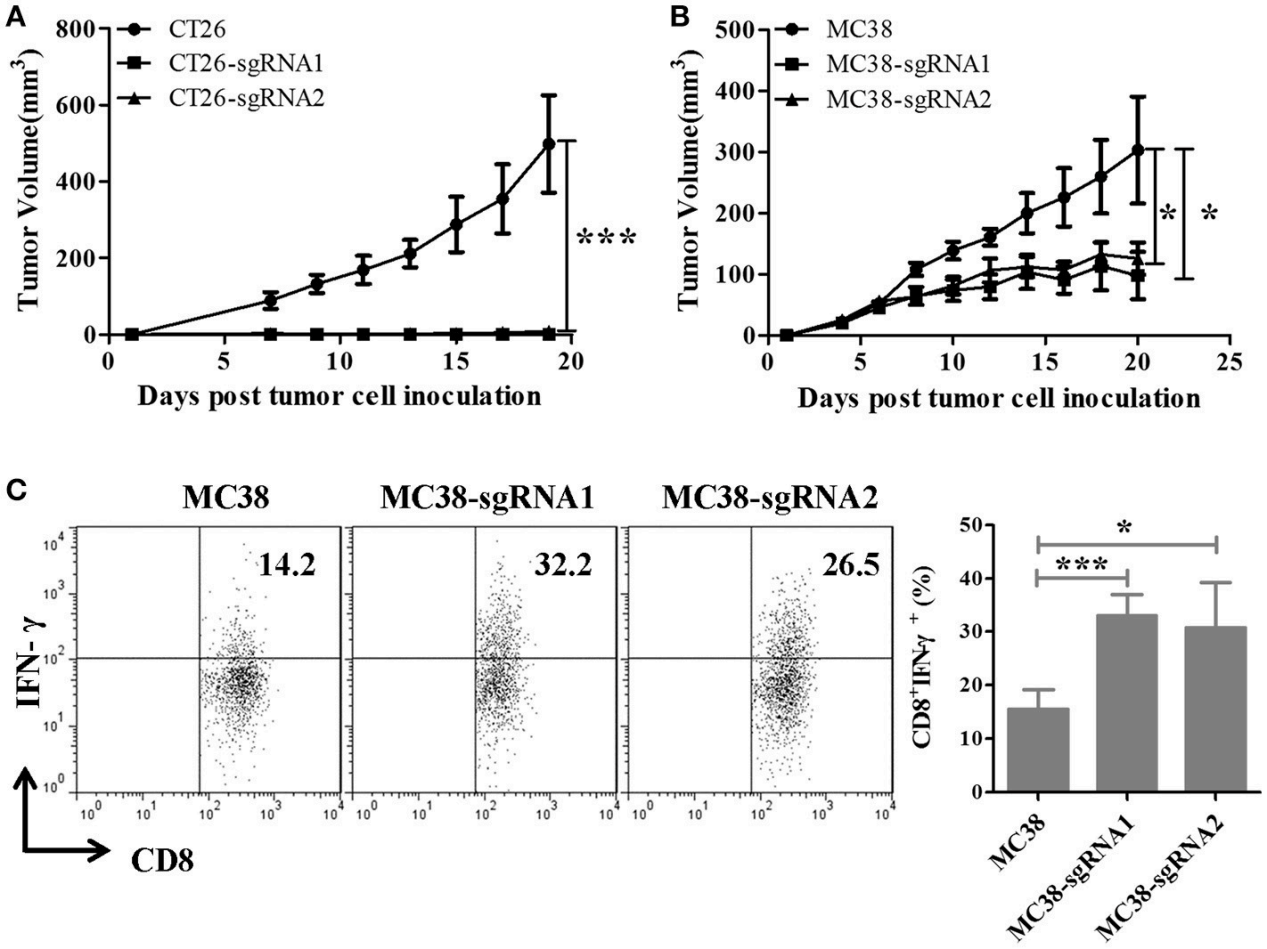
Knockout of intrinsic TIGIT remarkably impaired the tumorigenicity of murine colorectal cancer cells *in vivo*. **(A)** BALB/c mice were subcutaneously injected on the right back with 1 × 10^5^ syngeneic CT26 cells or the TIGIT KO cells (CT26-sgRNA1 or CT26-sgRNA2). **(B)** C57BL/6 mice were subcutaneously injected on the right back with 5 × 10^5^ syngeneic MC38 cells or the TIGIT KO cells (MC38-sgRNA1 and MC38-sgRNA2). Tumors were measured every two days using a digital caliper, and tumor volumes were calculated using the formula V = 1/2 × a (length) × b (width) × c (height). **(C)** MC38 tumor-infiltrating lymphocyte (TIL) were isolated and stimulated with 20 ng PMA and 1 μM ionomycin in the presense of protein transport inhibitor cocktail for 4 h. The frequency of IFN-γ-secreting CD8^+^ TIL cells were detected by FACS. Representative data are shown from three experiments conducted with 6–10 mice per group. Data are presented as mean values (± SEM). Statistical significance was determined by Student's *t*-test (^*^p < 0.05, ^***^p < 0.001).

Since the knockout of intrinsic expression of TIGIT in tumor cells did not affect cell proliferation and colony formation, the tumor growth ability was examined in mice. BABL/c mice were inoculated with CT26 or TIGIT KO CT26 cells, and C57BL/6 mice with MC38 or TIGIT KO MC38 cells. Compared with control CT26 cells, TIGIT knockout (CT26-sgRNA1 or CT26-sgRNA2) significantly inhibited the tumorigenicity (*p* < 0.001) (Figure 3A). Visible but very tiny tumors were generated at the beginning, and then regressed soon. When the mice with tumor regression were re-challenged using 5-fold amount of CT26, CT26-sgRNA1 or CT26-sgRNA2 cells, mice exhibited protective antitumor response (data not shown). Similar tumor inhibition along with a higher production of IFN-γ by tumor-infiltrating T cells were seen in C57BL/6 mice bearing TIGIT knockout MC38 cells (Figures 3B,C). Taken together, these results indicated that tumor-intrinsic TIGIT contributed to helping tumor growth in murine colorectal cancer models, and this effect might depend on the host immune response.

### Tumor-intrinsic TIGIT compromised the function of NK and CD8^+^ T cells

**Figure 4:**
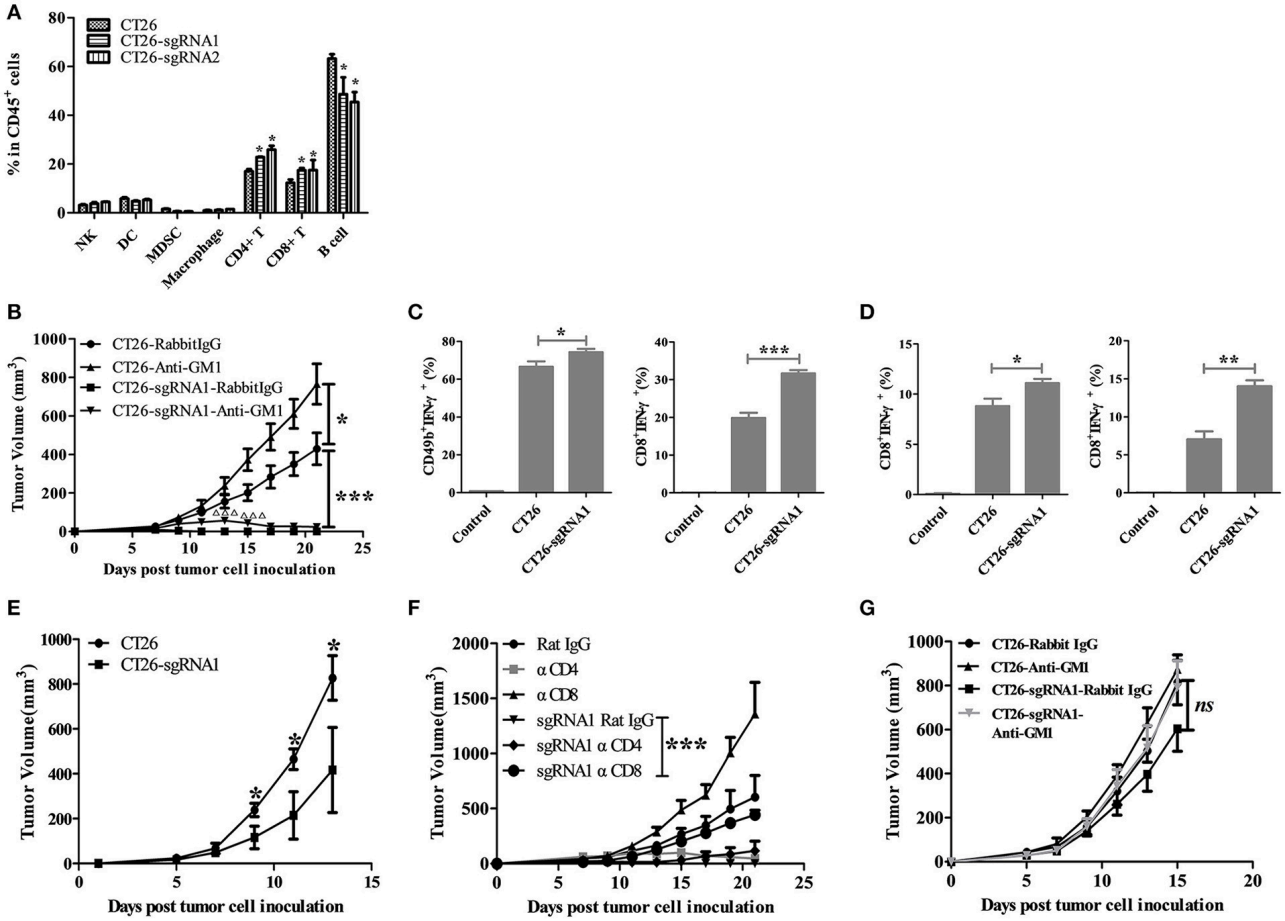
Tumor-intrinsic TIGIT compromised the function of NK and CD8^+^ T cells. **(A)** BALB/c mice were treated as in Figure 3A. Three weeks after tumor cell inoculation, splenocytes from tumor-bearing BALB/c mice were harvested (*n* = 3). Single cell suspensions were then stained with indicated antibodies, and the percentages of cell population were analyzed. **(B)** BALB/c mice were subcutaneously injected on the right back with 1 × 10^5^ syngeneic CT26 and CT26-sgRNA1 cells. Starting from the day before tumor cell inoculation, 250 μg anti-asialo-GM1antibody or rabbit IgG isotype control was injected *i.p*. every 4 days (*n* = 5). Tumor volumes were calculated as in Figure 3A (^*^p < 0.05, ^***^p < 0.001, significant differences between CT26-sgRNA1 tumor-bearing mice treated with or without anti-asialo-GM1were indicated by ^Δ^,^ΔΔΔ^*p* < 0.001). **(C)** Mice treated with rabbit IgG were sacrificed on day 21 after tumor cell inoculation, NK and CD8^+^ T cells were sorted from the spleen of tumor-bearing mice by MACS. The percentages of IFN-γ^+^ secreting NK cells and CD8^+^ T cells were detected by FACS (^*^p < 0.05, ^***^p < 0.001). **(D)** Mice treated with anti-asialo-GM1 antibody were sacrificed on day 21 after tumor cell inoculation, CD8^+^ T cells were sorted from the spleen (upper) and draining lymph node (dLN) (lower) by MACS. The percentages of IFN-γ^+^ secreting CD8^+^ T cells were detected by FACS. (*n* = 5). **(E)** BALB/c nude mice were injected with 1 × 10^5^ CT26 cells or CT26-sgRNA1 cells. (*n* = 5) **(F)** BALB/c mice were subcutaneously injected on the right back with 1 × 10^5^ syngeneic CT26 and CT26-sgRNA1 cells. Starting from the day before tumor cell inoculation, 200 μg CD8 depleting antibody (clone: YTS.169.4) or 250 μg CD4 depleting antibody (clone: GK1.5) was injected *i.p*. every 4 days. (*n* = 5). (**G**) BALB/c nude mice were injected with 1 × 10^5^ syngeneic CT26 cells or CT26-sgRNA1 cells, and treated with anti-asialo-GM1 antibody or control (*n* = 5). (^*^p < 0.05, ^**^p < 0.01).

To figure out which immune cell subsets contribute to this tumor growth inhibition process, the percentage changes of different immune cell populations of splenocytes from tumor-bearing mice were examined. The percentages of CD4^+^ T and CD8^+^ T cells were increased in TIGIT KO CT26 cells bearing BABL/c mice (Figure 4A). Considering that most of the studies reported that TIGIT function via NK cells, we proposed that NK and T cells might both be inhibited by TIGIT on CT26 tumor cells. To verify this, the TIGIT KO tumor model was established in NK depleting mice. Consistent with results in Figure 3, TIGIT knockout could dramatically impair the tumorigenicity in CT26-sgRNA1 group with rabbit IgG control antibody (Figure 4B, CT26-Rabbit IgG vs. CT26-sgRNA1-Rabbit IgG, *p* < 0.001). Without NK cells, TIGIT KO tumors could grow at the beginning and then regressed, suggesting that NK cells might work at early phase and T cells at latter phase (Figure 4B, CT26-sgRNA1-Rabbit IgG vs. CT26-sgRNA1-Anti-asialo-GM1, *p* < 0.001 on day 13 and 15). The tumor volumes of individual tumor bearing mice were also displayed. (Figure S5). To confirm the function of these immune cells, NK cells and CD8^+^ T cells were sorted from tumor bearing mice by magnetic activated cell sorting (MACS). The sorted cells were stimulated by PMA and ionomycin, and the secretion of IFN-γ was detected by intracellular cytokine staining assay. Compared with CT26-Rabbit IgG group, NK cells and CD8^+^ T cells sorted from CT26-sgRNA1-Rabbit IgG group gains potent ability to secrete IFN-γ, the percentage of IFN-γ^+^ NK cells increased about 8%, the percentage of IFN-γ^+^ CD8^+^ T cells augmented about 1.5 folds (Figure 4C, Figure S6A). Further, to explore the effects of tumor intrinsic TIGIT on CD8^+^ T cells without the existence of NK cells. CD8^+^ T cells were sorted from the spleen and draining lymph node of CT26 and CT26-sgRNA1 bearing mice treated with anti-asialo-GM1, higher production by sorted CD8^+^ T cells from CT26-sgRNA1 bearing mice coincided with significant tumor inhibition (Figure 4D, Figure S6B). These results indicated that TIGIT on tumor cells could inhibit the function of both NK and CD8^+^ T cells.

### The tumor promoting effects of tumor-intrinsic TIGIT are mainly dependent on CD8^+^ T cells

Further, we established BABL/c nude and CD8^+^ T cells depleting model to verify the role of CD8^+^ T cells in the process of tumor inhibition by TIGIT knockout. The tumor growth inhibition led by TIGIT knockout was significantly rescued in BABL/c nude mice, indicating that impairment of T cell function might be mediated by TIGIT on tumor cells. Meanwhile, in BABL/c nude mouse model, TIGIT knockout could not entirely rescue tumor growth, suggesting that tumor-intrinsic TIGIT may suppress other anti-tumor immune cells (probably NK cells) independently of T cells (Figure 4E). To further determine the potential role of CD4^+^ and CD8^+^ T cells, they were depleted, respectively, in CT26-sgRNA1 bearing BABL/c mice. Compared with the control group, depletion of CD8^+^ T cells completely abolished the tumor growth inhibition induced by TIGIT knockout (Figure 4F), while CD4^+^ T cell depletion exhibited weaker impact on tumor growth. Finally, we established a model that lacks both T cells and NK cells by depleting NK cells on BABL/c nude mice, the tumor growth inhibition led by TIGIT knockout was significantly rescued in the absence of T cells and NK cells, without significant difference between the CT26-sgRNA1 groups treated with control or anti-asialo-GM-1 (Figure 4G). Collectively, these results indicated that tumor-intrinsic TIGIT could promote tumor growth mainly by suppressing the function of CD8^+^ T cells.

Previous studies have demonstrated that TIGIT primarily exert its immune suppressive function through interaction with PVR. Interestingly, except that TIGIT was reported to be expressed on NK and T cells, the expression of PVR was also observed on CD4^+^ T cells, CD8^+^ T cells and NK cells (Figure S7), indicating the function of these cells might be suppressed by TIGIT on tumor cells via interaction with PVR.

### TIGIT blockade could inhibit tumor growth through blocking tumor intrinsic TIGIT

**Figure 5:**
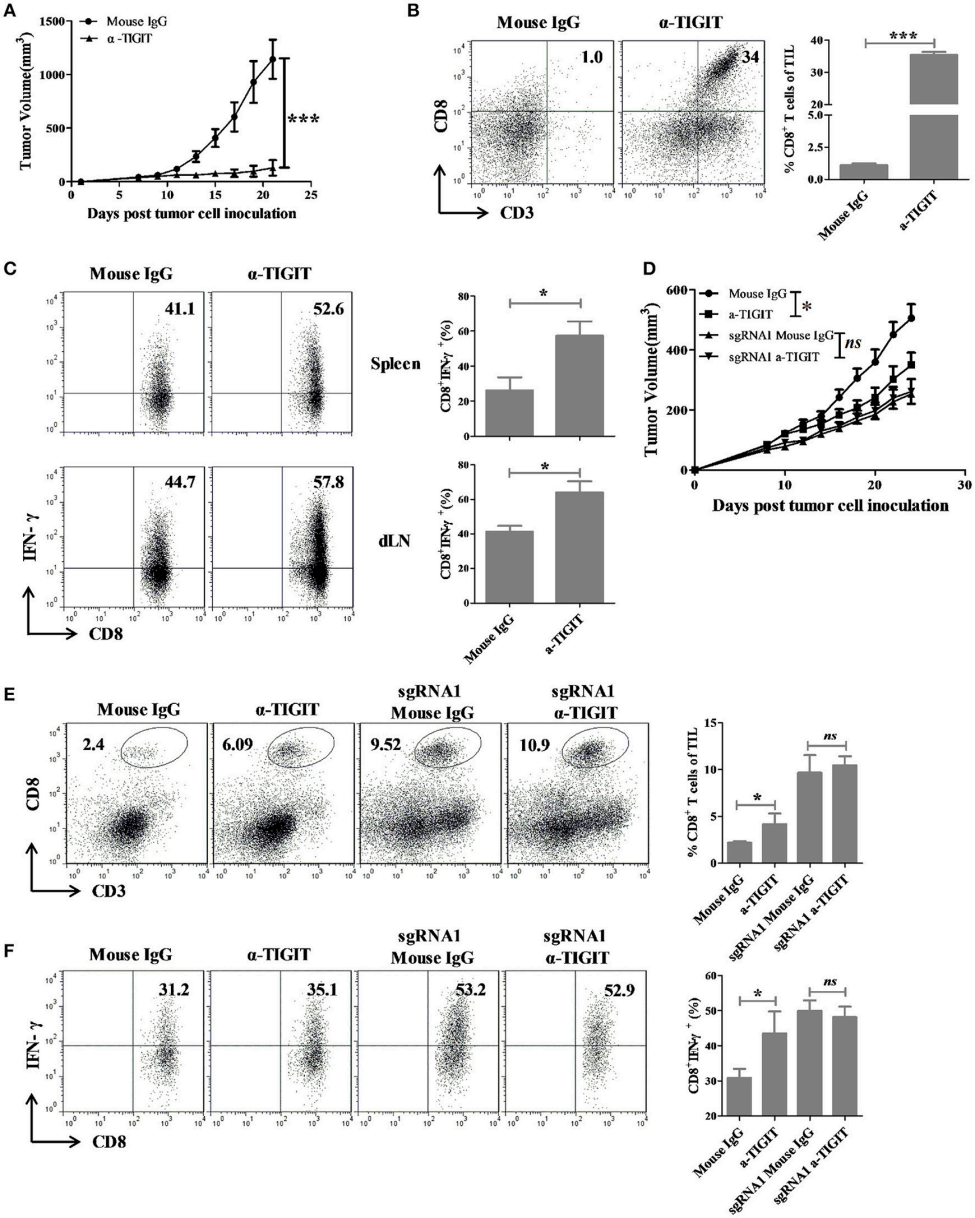
TIGIT blockade elicit anti-tumor effects in colorectal cancer mouse model through blocking TIGIT on tumor cells. **(A)** BALB/c mice were subcutaneously injected in the right back with 1 × 10^5^ syngeneic CT26 cells. Seven days later, mice bearing tumors of 50–100 mm^3^ were randomly grouped and treated with α-TIGIT (200 μg), isotype control (200 μg) by intraperitoneal injection every 3 days for 2 weeks. **(B,C)** Mice were sacrificed on day 21 after treatment for 2 weeks, **(B)** tumors were digested into single cell suspension and the percentages of infiltrating CD8^+^ T cells were detected by FACS. **(C)** Spleen and draining lymph node were digested into single cell suspension and stimulated with 20 ng PMA and 1 μM ionomycin in the presence of protein transport inhibitor cocktail for 4 h. The percentages of IFN-γ^+^ secreting CD8^+^ T cells were detected by FACS. Statistical significance was determined by Student's *t*-test (*n* = 5, ^*^p < 0.05, ^***^p < 0.001). **(D)** C57BL/6 mice were subcutaneously injected in the right back with 1 × 10^6^ syngeneic MC38 cells. Ten days later, mice bearing tumors of 50–100 mm^3^ were randomly grouped and treated with α-TIGIT (200 μg) or isotype control (200 μg) by intraperitoneal injection every 3 days for 2 weeks. **(E)** Mice were sacrificed after treatment for 2 weeks, tumors were digested into single cell suspension and the frequency of infiltrating CD8^+^ T cells were detected by FACS. **(F)** TIL were separated and restimulated with 20 ng/mL PMA and 1 μM Ion in the presence of BD GolgiPlug for 4 h, and the frequency of IFN-γ-secreting CD8^+^ T cells were detected by FACS. Statistical significance was determined by Student's *t*-test (*n* = 4–6, ^***^p < 0.001).

Since TIGIT knockout could inhibit the tumor growth *in vivo*, it is necessary to investigate whether TIGIT blockade has anti-tumor effects. Anti-TIGIT antibody and recombinant mouse PVR protein were used to treat the colorectal tumor CT26 bearing mice. The tumors in PVR and α-TIGIT treated groups were significantly inhibited compared to that of isotype control group (Figure 5A, α-TIGIT vs. isotype control, Figure S8A, *p* < 0.001; PVR vs. isotype control, *p* < 0.01). Blockade of TIGIT with α-TIGIT significantly increased the CD8^+^ T cells infiltration into the tumor (Figure 5B, Figure S8B). Moreover, IFN-γ production by CD8^+^ T cells of tumor-draining lymph node and spleen increased along with the inhibition of tumor growth caused by TIGIT blockade with α-TIGIT (Figure 5C) but not by PVR protein (Figure S8C).

To further distinguish the effects of TIGIT blockade on tumor-TIGIT and immune cell-TIGIT, MC38 and TIGIT KO MC38 mouse model were established and mice were treated with TIGIT antibody or isotype control. Antibody-mediate TIGIT blockade could significantly inhibit tumor growth in MC38-bearing mice, but not in MC38-sgRNA1 bearing mice (Figure 5D). Besides, TIGIT blockade could significantly increase the percentage and IFN-γ production of tumor infiltrating CD8^+^ T cells along with tumor inhibition in MC38 but not in MC38-sgRNA1 bearing mice (Figures 5E,F). These results revealed that antibody-mediate TIGIT blockade could inhibit tumor growth through blocking TIGIT expressed on tumor cells.

## Discussion

Considering the lower response rates of PD-1/PD-L1 blockade therapy in colorectal cancer patients, novel targets and combinations are urgently needed (36–40). Among the alternative immune checkpoint molecules, TIGIT is reported to be a promising target for cancer immunotherapy, but its function remains largely unknown(41). Here, our study updates the current knowledge of the immune checkpoint TIGIT in several aspects and identifies the vital role of TIGIT in tumor cells.

Firstly, we demonstrated that tumor cells could intrinsically express TIGIT for the first time, while TIGIT was previously reported to be an inhibitory molecule of immune cells (19–21). Until now, only the immune checkpoint receptor molecule PD-1 has been reported to express on melanoma cells, but PD-1 is not uniformly expressed on all melanoma cells but restricted to a very small subpopulation (35). By RT-PCR and flow cytometry, we verified that TIGIT widely expressed on different tumor cell types including colon cancer, breast cancer, melanoma, and lung carcinoma cell lines. Compared to the expression pattern of PD-1 on melanoma cells, TIGIT was intrinsically expressed on tumor cells in a broader range. Although TIGIT expression occurs mostly on CD45^+^ immune cells in human colorectal cancer, the obvious expression of TIGIT was observed on CD45^−^ cells in some cases. The reason for the expression difference is still unclear, and the expression of TIGIT on colorectal cancers after certain treatments or on other cancer types remains to be investigated.

Secondly, different from PD-1 on melanoma cells, intrinsic TIGIT does not affect the proliferation of tumor cells *in vitro*. These results were similar to the effects of another immune co-stimulatory molecule LIGHT over-expressed on tumor cells (42). These functions are absolutely different from the effects of TIGIT on T cells in transmitting inhibitory signal and mediating tumor evasion through the ITIM motif by ligation with PVR (43–45). Previous studies have demonstrated that melanoma-intrinsic PD-1 could drive pro-tumorigenic effects by interaction with host- and/or tumor cell expressed PD-L1. However, our study showed that tumor-intrinsic TIGIT did not inhibit the function of tumor cells, which may due to the TIGIT-PVR interaction did not result in any inhibitory consequences *in vitro*, or other unknown signals to compensate.

Further, our results demonstrated that tumor-intrinsic TIGIT could maintain tumor growth in colorectal cancer models. TIGIT knockout CT26 tumors could grow at the beginning when NK cells were depleted, but gradually regressed along with the activation of adaptive immunity. In BABL/c nude mice and CD8^+^ T cell depleting mice, the tumor growth inhibition caused by TIGIT knockout could not be entirely rescued in the absence of CD8^+^ T cells. This implies there might be other factors which affect the growth of TIGIT-expressing tumors. According to the results in Figure 2 that tumor-intrinsic TIGIT did not affect the growth and colony formation of tumor cells, we hypothesized that the anti-tumor effects of other immune cells independently of CD8^+^ T cells might be inhibited. Further, the results that there are no growth differences between CT26 tumors and TIGIT KO CT26 tumors without the existence of both NK cells and CD8^+^ T cells verifies our hypothesis. In conclusion, tumor-intrinsic TIGIT could suppress the function of both NK cells and CD8^+^ T cells. Our results on tumor-intrinsic TIGIT were similar to the current studies that TIGIT on NK and CD8^+^ T cells could suppress their anti-tumor effects.

Besides, we also confirmed the expression of ligand PVR on NK and T cells by flow cytometry. Together, these results provide possibility that tumor cells exert immunosuppressive role on NK and CD8^+^ T cells through the interaction of tumor-TIGIT and immune cells-PVR. It has been reported that PVR harbored an immune-receptor tyrosine-based inhibition motif (ITIM) in its cytoplasmic tail (46). Ligation with TIGIT could trigger intracellular phosphorylation of PVR expressed on DC cells, enhanced the secretion of IL-10, decreased the secretion of IL-12 and therefore indirectly inhibit the function of T cells(20, 47). Therefore, we propose that tumor intrinsic TIGIT may deliver inhibitory signals to CD8^+^ T cells and NK cells by engaging with PVR.

PD-1 was widely accepted as the first choice for immune checkpoint based cancer immunotherapy. In the current study, we found that TIGIT was overexpressed in tumor tissues and expressed higher than PD-1 in most of the colorectal cancer samples. To confirm whether TIGIT could be considered as the target for immunotherapy, we observed that TIGIT blockade using antibody or recombinant mouse PVR protein could also suppress tumor growth, augment tumor-infiltrating lymphocytes and boost anti-tumor immune response in CT26 mice model. Although the PVR protein slightly increased the proportion of CD8^+^ T cells in the tumor site compared with α-TIGIT, the PVR protein could significantly inhibit the growth of CT26 tumors. To the difference from α-TIGIT, it might be that the affinity of the PVR protein is inferior to that of α-TIGIT (verified by flow cytometry). Whether PVR proteins exert tumor inhibition by interacting with other cells types remains investigated. The blockade effects on TIGIT expressed on tumor cells and immune cells has not been well distinguished. Consistent with the results of TIGIT antibody (1B4) (48), TIGIT antibody (1G9) could also significantly inhibit the growth of MC38 tumors in WT mice. However, no obvious growth inhibition was observed on TIGIT KO MC38 tumors on WT mice, and this suggested that TIGIT expressed on immune cell did not affect the growth of MC38 tumors (Figure 5D). The results parallel with the report that there was no significant changes of MC38 tumors growth in WT mice compared to TIGIT^−/−^ mice without TIGIT expression on immune cells (49). Therefore, the antibody-mediated TIGIT blockade could inhibit MC38 tumor growth through blocking TIGIT expressed on tumor cells.

Taken together, our study for the first time discovered that tumor cells could intrinsically express TIGIT. Tumor-intrinsic TIGIT might perform its suppressing function differently from that on immune cells. Our findings elucidated that tumor-intrinsic TIGIT could help the tumor to grow by suppressing the function of NK and CD8^+^ T cells, which can be restored by TIGIT antibody or PVR blockade. Therefore, TIGIT could be a potential target for immunotherapy of colorectal cancer.

## Author contributions

X-MZ and Y-FG conceived the research and designed the experiments; X-MZ and W-QL performed the majority of experiments with critical support from Y-HW, LH, H-FW, W-SZ, and W-JZ; X-GC provided the patient samples; X-MY and Y-FG proposed important concepts; X-MZ wrote the first draft of the manuscript; all authors contributed to manuscript revision, read, and approved the submitted version.

### Conflict of interest statement

The authors declare that the research was conducted in the absence of any commercial or financial relationships that could be construed as a potential conflict of interest.

## Supplementary material

Figure S1 TIGIT expression on colorectal tumor and matched peri-tumor tissues. Colorectal tissues were processed into single cell suspension, stained with anti-CD45 and anti-TIGIT antibody, the expression of TIGIT was analyzed by flow cytometry and the frequencies of CD45^+^ TIGIT^−^ with the isotype control percent subtracted were counted.

Figure S2 TIGIT and PD-1 were expressed in different murine tumor cell lines in mRNA level. RT-PCR expression analysis of TIGIT and PD-1 mRNA by murine tumor cell lines.

Figure S3 TIGIT expression on murine tumor cell lines, related to Figure 1. Flow cytometry analysis of TIGIT expression by anti-TIGIT (Clone: 1G9).

Figure S4 PVR and PD-L1 expression on TIGIT knockout (KO) CT26 and MC38 cell lines. Flow cytometry analysis of PD-L1 and PVR expression of the parental cells lines CT26 and MC38 (red line), and the TIGIT knockout (KO) cell lines CT26-sgRNA1, MC38-sgRNA1 (blue line), CT26-sgRNA2, MC38-sgRNA2 (green line), the gray-shaded histogram represents the isotype control.

Figure S5 Tumor volumes of individual tumor bearing mice, related to Figure 4B. BALB/c mice were subcutaneously injected on the right back with 1 × 10^5^ syngeneic CT26 and CT26-sgRNA1 cells. Starting from the day before tumor cell inoculation, 250 μg anti-asialo-GM1antibody or rabbit IgG isotype control was injected *i.p*. every 4 days (*n* = 5).

Figure S6 The potency of sorted NK cells or CD8^+^ T cells to secrete IFN-γ, related to Figures 4C,D. **(A)** Representative dot plots of IFN-γ^+^ secreting NK cells (upper) and CD8^+^ T cells (lower). NK and CD8^+^ T cells were sorted from the spleen of tumor-bearing mice treated with rabbit IgG by MACS. **(B)** Representative dot plots of IFN-γ^+^ secreting CD8^+^ T cells. CD8^+^ T cells were sorted from the spleen (upper) and draining lymph node (dLN) (lower) of tumor-bearing mice treated with anti-asialo-GM1 antibody by MACS.

Figure S7 PVR expression on immune cells. Representative flow cytometry histogram of PVR expression on CD4^+^ T cells, CD8^+^ T cells and NK cells (CD45^+^CD3^−^CD49b^+^). The gray-shaded histogram represents the isotype control.

Figure S8 TIGIT blockade elicit anti-tumor effects in colorectal cancer mouse model. **(A)** BALB/c mice were subcutaneously injected in the right back with 1 × 10^5^ syngeneic CT26 cells. Seven days later, mice bearing tumors of 50–100 mm^3^ were randomly grouped and treated with normal saline (NS) or PVR protein (200 μg) by intraperitoneal injection every 3 days for two weeks. **(B,C)** Mice were sacrificed on day 21 after treatment for two weeks, **(B)** tumors were digested into single cell suspension and the percentages of infiltrating CD8^+^ T cells were detected by FACS. **(C)** Spleen and draining lymph node were digested into single cell suspension and stimulated with 20 ng PMA and 1 μM ionomycin in the presence of protein transport inhibitor cocktail for 4 h. The percentages of IFN-γ^+^ secreting CD8^+^ T cells were detected by FACS. Statistical significance was determined by Student'fs *t*-test (*n* = 5, ^**^*p* < 0.01).
