# Supplementary figures and images for: Intrinsic Expression of Immune Checkpoint Molecule TIGIT Could Help Tumor Growth in vivo by Suppressing the Function of NK and CD8+ T Cells
Source: Front Immunol. 2018 Nov 29;9:2821. doi: 10.3389/fimmu.2018.02821 (PMC6281988; doi:10.3389/fimmu.2018.02821)

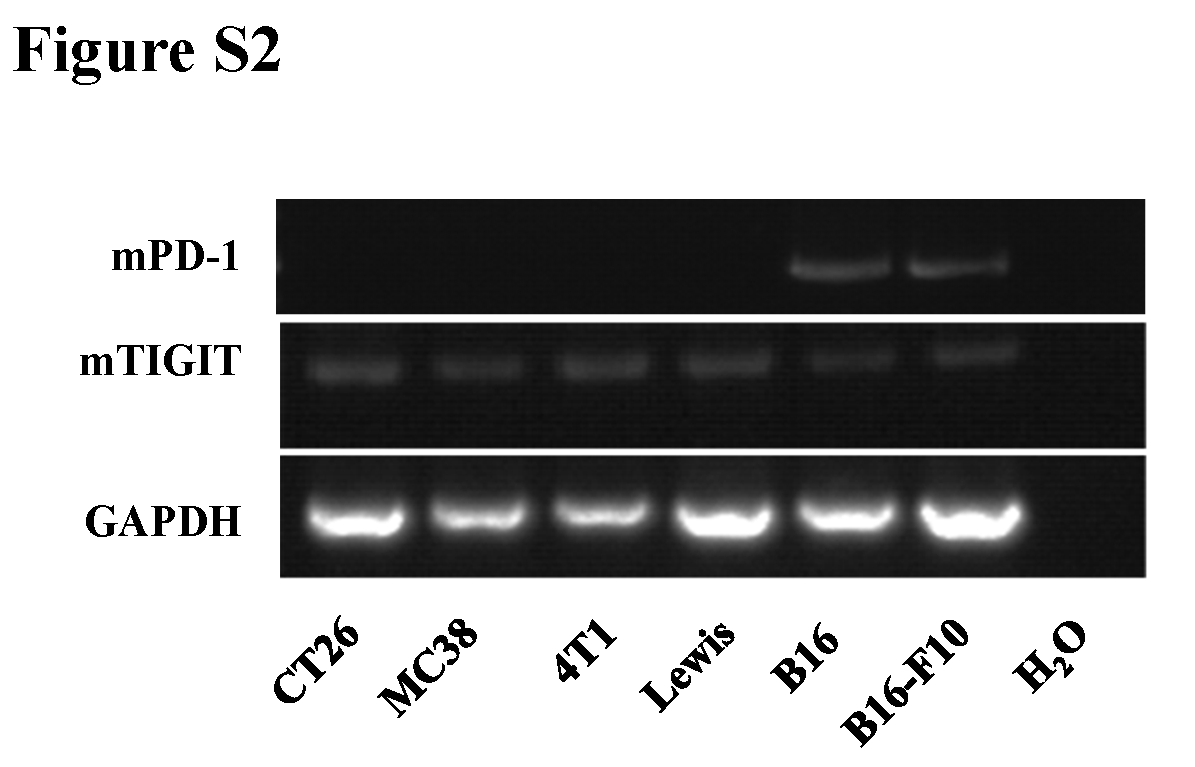

Supplement: Figure S2 — TIGIT and PD-1 were expressed in different murine tumor cell lines in mRNA level. RT-PCR expression analysis of TIGIT and PD-1 mRNA by murine tumor cell lines. [file Image_2.TIFF]

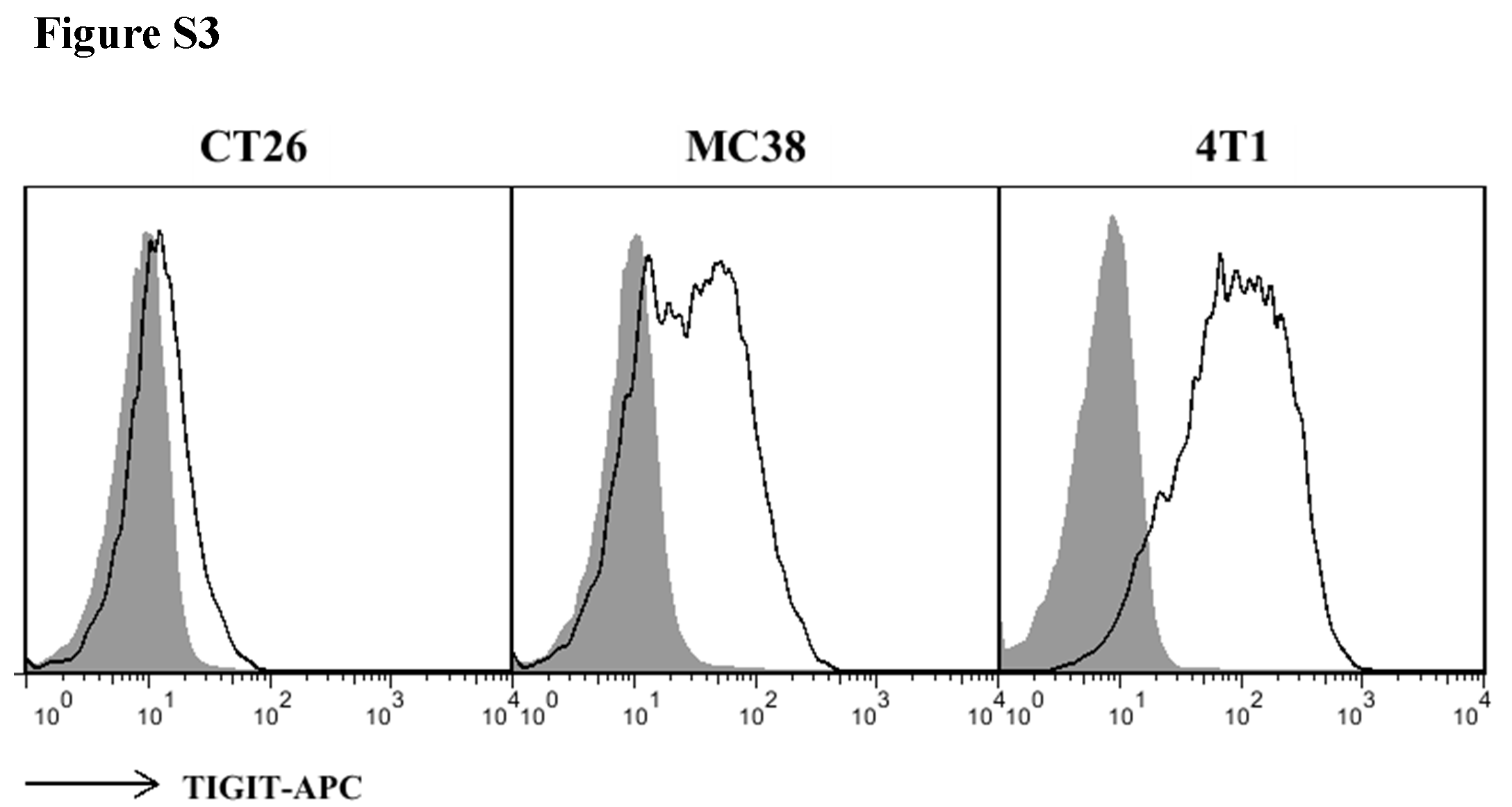

Supplement: Figure S3 — TIGIT expression on murine tumor cell lines, related to Figure 1. Flow cytometry analysis of TIGIT expression by anti-TIGIT (Clone: 1G9). [file Image_3.TIFF]

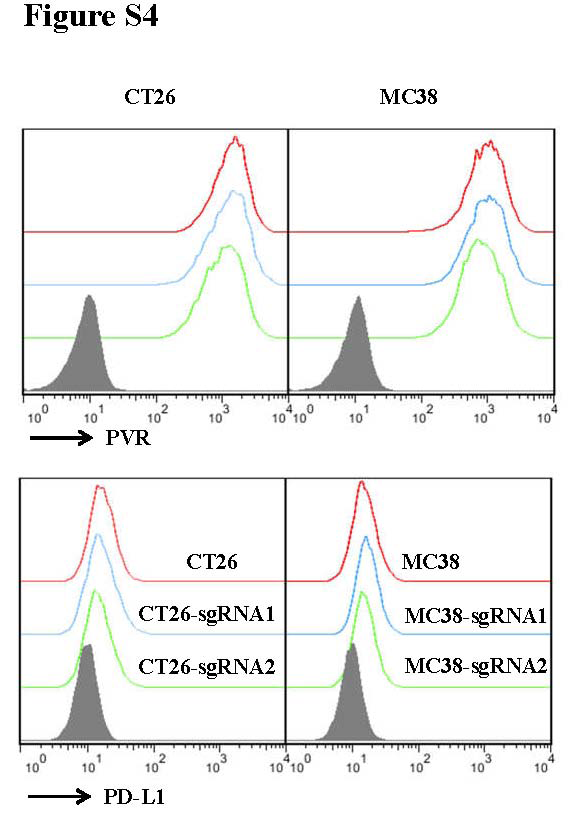

Supplement: Figure S4 — PVR and PD-L1 expression on TIGIT knockout (KO) CT26 and MC38 cell lines. Flow cytometry analysis of PD-L1 and PVR expression of the parental cells lines CT26 and MC38 (red line), and the TIGIT knockout (KO) cell lines CT26-sgRNA1, MC38-sgRNA1 (blue line), CT26-sgRNA2, MC38-sgRNA2 (green line), the gray-shaded histogram represents the isotype control. [file Image_4.TIFF]

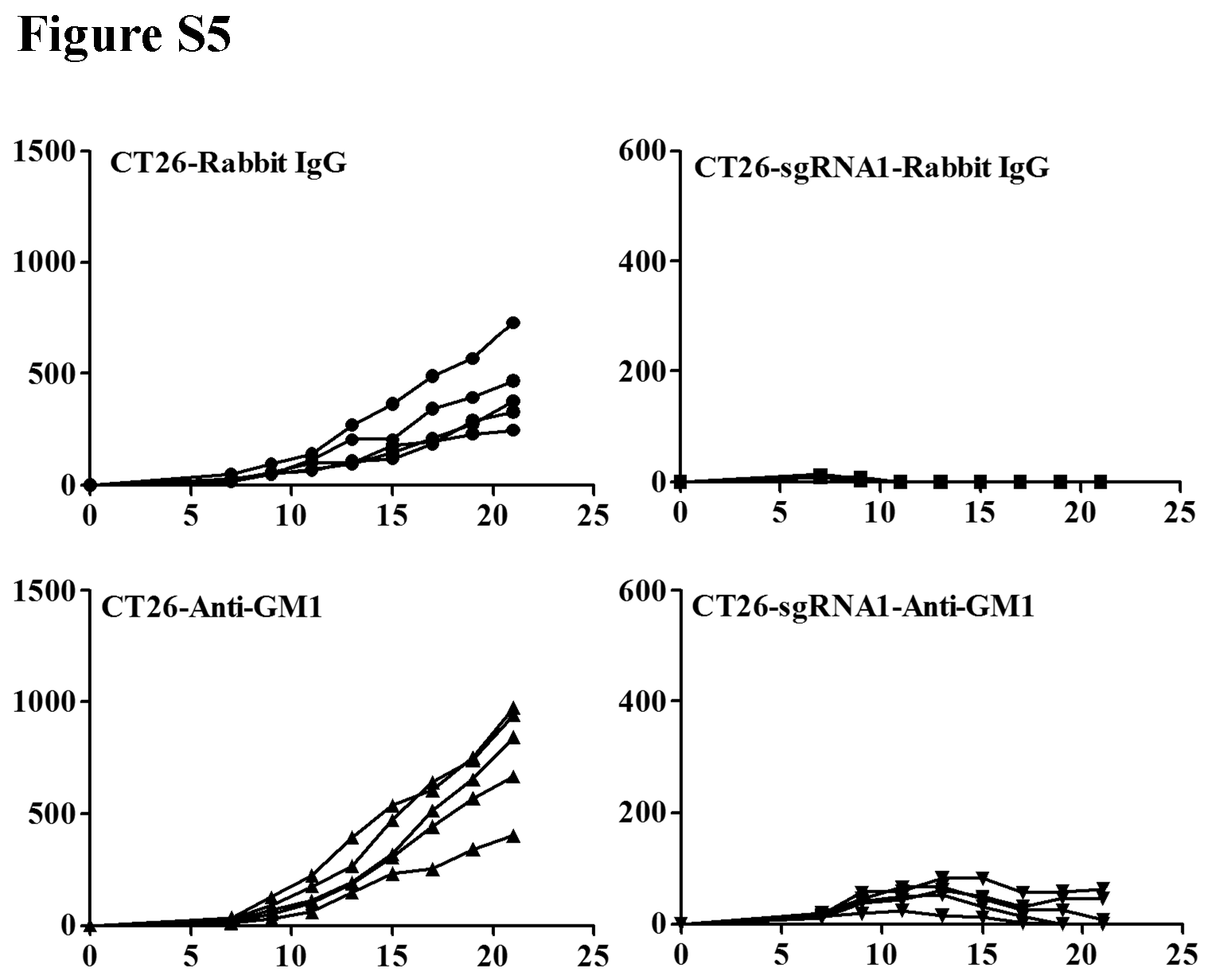

Supplement: Figure S5 — Tumor volumes of individual tumor bearing mice, related to Figure 4B. BALB/c mice were subcutaneously injected on the right back with 1 × 10^5^ syngeneic CT26 and CT26-sgRNA1 cells. Starting from the day before tumor cell inoculation, 250 μg anti-asialo-GM1antibody or rabbit IgG isotype control was injected i.p. every 4 days (n = 5). [file Image_5.TIFF]

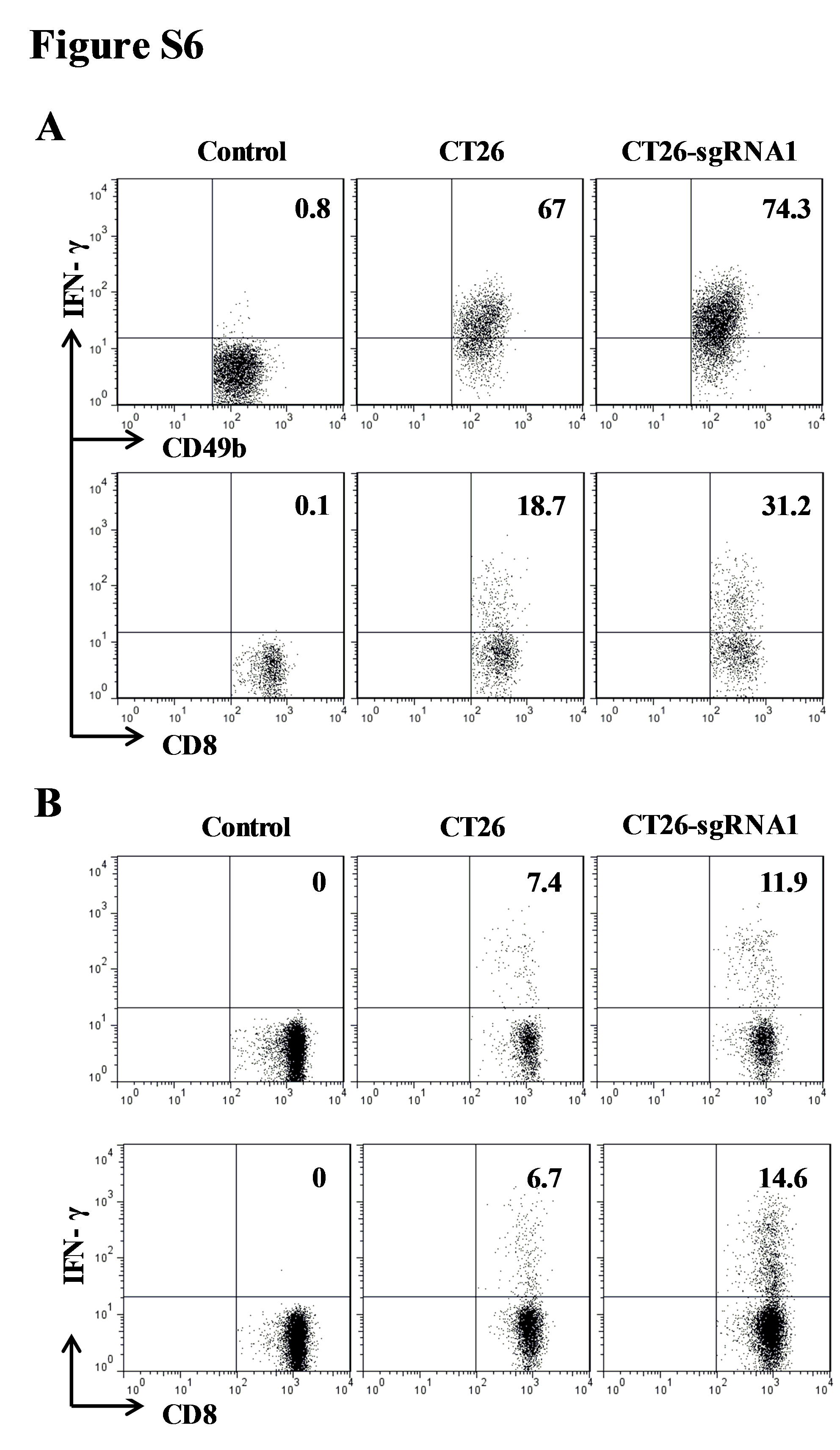

Supplement: Figure S6 — The potency of sorted NK cells or CD8+ T cells to secrete IFN-γ, related to Figures 4C,D. (A) Representative dot plots of IFN-γ+ secreting NK cells (upper) and CD8+ T cells (lower). NK and CD8+ T cells were sorted from the spleen of tumor-bearing mice treated with rabbit IgG by MACS. (B) Representative dot plots of IFN-γ+ secreting CD8+ T cells. CD8+ T cells were sorted from the spleen (upper) and draining lymph node (dLN) (lower) of tumor-bearing mice treated with anti-asialo-GM1 antibody by MACS. [file Image_6.TIFF]

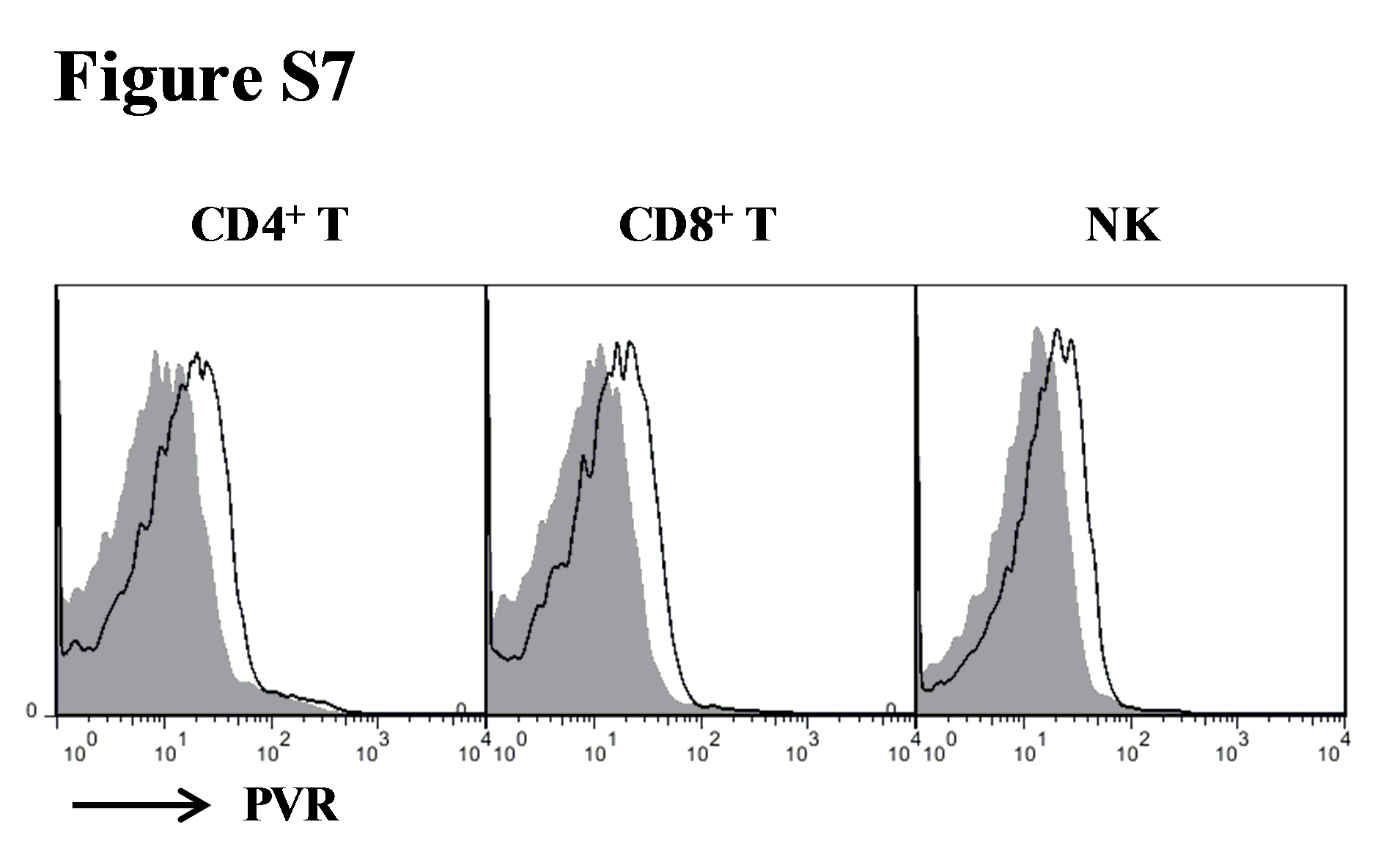

Supplement: Figure S7 — PVR expression on immune cells. Representative flow cytometry histogram of PVR expression on CD4+ T cells, CD8+ T cells and NK cells (CD45+CD3−CD49b+). The gray-shaded histogram represents the isotype control. [file Image_7.TIFF]
